# Sense of Control and Cognitive Aging: The Mediating Role of Risk and Protective Factors

**DOI:** 10.1093/geroni/igaf122.4006

**Published:** 2025-12-31

**Authors:** Nathan Merin, Kylie Schiloski, Margie Lachman

**Affiliations:** Brandeis University, Burlingame, California, United States; Brandeis University, Waltham, Massachusetts, United States; Brandeis University, Waltham, Massachusetts, United States

## Abstract

Sense of control is a psychosocial factor that is known to be associated with engagement in protective health behaviors and cognitive and physical health outcomes in middle age and later life. The present study tested whether sense of control was related to risk and protective factors contributing to brain health measured by the McCance Brain Care Score (BCS), an index of modifiable physical (blood pressure, blood glucose, cholesterol, Body Mass Index), lifestyle (nutrition, exercise, smoking status, sleep, alcohol consumption), and psychosocial factors (stress, relationships, purpose in life). We hypothesized that the BCS would mediate the relationship between sense of control and 9-year changes in cognitive and physical health. Data were collected from the second (M2) and third (M3) waves of the Midlife in the United States (MIDUS) Study and biomarker sub-study. Results showed that the relationships between sense of control and 9-year residual change in episodic memory, functional health, and chronic conditions were all mediated by the BCS, with each model controlling for age, sex, race, and level of education. The BCS also mediated the relationship between sense of control and level of performance 9-years later for executive functioning. These findings demonstrate that those with a higher sense of control were more likely to have a higher brain care score, which in turn was related to a greater maintenance of specific cognitive and physical health outcomes. This work suggests that promoting a sense of control shows potential for enhancing brain health and maintaining favorable cognitive and physical health.

